# Towards secure IoT networks: A comprehensive study of metaheuristic algorithms in conjunction with CNN using a self-generated dataset

**DOI:** 10.1016/j.mex.2024.102747

**Published:** 2024-05-04

**Authors:** Vandana Choudhary, Sarvesh Tanwar, Tanupriya Choudhury, Ketan Kotecha

**Affiliations:** a Amity Institute of Information Technology, Amity University, Noida 201313, India; b Research Professor, CSE Department, Graphic Era Deemed to be University, Dehradun, Uttarakhand 248002, India; c Adjunct Professor, CSE Department, Symbiosis Institute of Technology, Symbiosis International (Deemed University) (SIU), Lavale Campus, Pune, Maharashtra 412115, India; d Symbiosis Centre for Applied Artificial Intelligence (SCAAI), Symbiosis International (Deemed University) (SIU), Symbiosis Institute of Technology, Lavale Campus, Pune 412115, India

**Keywords:** IoT security, Self-generated dataset, Metaheuristic algorithms, Convolutional neural network, CNN coupled with Metaheuristic Algorithms for Intrusion Detection in the Internet of Things

## Abstract

The Internet of Things (IoT) has radically reformed various sectors and industries, enabling unprecedented levels of connectivity and automation. However, the surge in the number of IoT devices has also widened the attack surface, rendering IoT networks potentially susceptible to a plethora of security risks. Addressing the critical challenge of enhancing security in IoT networks is of utmost importance. Moreover, there is a considerable lack of datasets designed exclusively for IoT applications. To bridge this gap, a customized dataset that accurately mimics real-world IoT scenarios impacted by four different types of attacks—blackhole, sinkhole, flooding, and version number attacks was generated using the Contiki-OS Cooja Simulator in this study. The resulting dataset is then consequently employed to evaluate the efficacy of several metaheuristic algorithms, in conjunction with Convolutional Neural Network (CNN) for IoT networks.

• The proposed study's goal is to identify optimal hyperparameters for CNNs, ensuring their peak performance in intrusion detection tasks.
• This study not only intensifies our comprehension of IoT network security but also provides practical guidance for implementation of the robust security measures in real-world IoT applications.

## Graphical abstract

**Figure:**
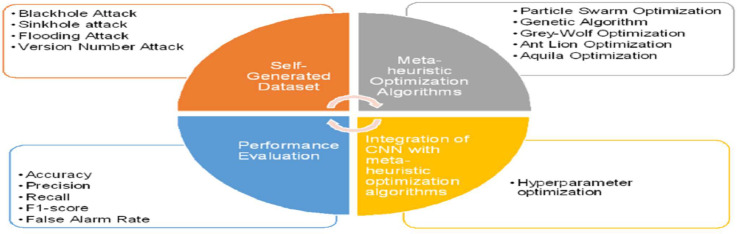

Specifications table

**Table:** 

Subject area:	Computer Science
More specific subject area:	Internet of Things
Name of your method:	CNN coupled with Metaheuristic Algorithms for Intrusion Detection in the Internet of Things
Name and reference of original method:	N.A.
Resource availability:	Data will be made available on request

**Method details**

## Introduction

IoT, a network which is formed by connecting physical devices with the aid of the Internet, enables devices to collect and exchange information without human involvement. The advent of innovative sensor technology, wireless communication, cloud computing, etc., has made the deployment of IoT solutions across vast array of domains economical and easy. As a result, a number of organizations and companies have decided to integrate IoT technology into their services. This integration of IoT devices has led to substantial achievement not only for individuals, households, and communities but also for the production sector, which in turn provides high-quality, convenient services, streamlined procedures, and inventive applications. The adoption of IoT has not only been embraced by individuals but also by governmental and private organizations for purposes such as productivity improvement, operation enhancement as well as the augmentation of the standard of living [[1], [2], [3]].

The introduction of IoT technology to the world markets has given rise to a fundamental risk issue - the lack of a proper security solution used to protect sensitive data and critical operations across IoT applications [[4], [5], [6]]. Cybercriminals are constantly honing their expertise, while traditional security measures encounter difficulty in staying on top of the latest trends, which reduces their effectiveness. Innovative and cybersecurity solutions introduced are the only apt ways to mitigate this gap and preserve the IoT networks from the ever-changing threat environment. Thus, the solution is to establish and implement an efficient intrusion detection system [[7], [8], [9]] that can be used for IoT environments. IoT-specific datasets designed for IoT applications and optimal tuning of the model's hyperparameters for machine learning and deep learning algorithms are the primary challenges towards efficient IoT IDSs development. The development and assessment of intrusion detection models for IoT environments have been constrained by the unavailability of IoT-specific datasets and this has in turn hindered the accurate detection and eradication of security threats.

Through devising robust intrusion detection methods for IoT networks, this research work will address these challenges. Our specific focus is on developing a customized dataset intended to mimic real-world IoT scenarios impacted by different types of security attacks. In addition, a range of metaheuristic algorithms [[10], [11], [12], [13]], such as PSO [14], GA [15], GWO [16], ALO [17], and AO [18], will be tested alongside CNNs [[19], [20]]. The goal is to find and tweak the hyperparameters that help CNNs work at their best and increase the accuracy and effectiveness of intrusion detection in IoT networks.

## Background study

The explosive expansion of IoT devices has propelled at the same time the IoT networks into a world that is full of security threats. Existing studies have concluded the need for IoT-specific datasets that sufficiently represent real-world scenarios of IoT, both benign and anomalous. These datasets serve as the backbone for training and evaluating intrusion detection models, enabling accurate threat identification and mitigation. However, the dearth of tailored IoT-specific datasets poses a significant impediment, limiting the scope and effectiveness of intrusion detection mechanisms in IoT networks [21,22]. Furthermore, various metaheuristic algorithms, such as PSO, GA, GWO, ALO, and AO, have been explored in various domains, but their application to optimize hyperparameters for machine and deep learning algorithms, particularly CNNs for intrusion detection in IoT environments presents a promising avenue. These algorithms offer the potential to fine-tune CNN hyperparameters, enhancing their accuracy and efficacy in identifying security threats within IoT networks.

The field of metaheuristic algorithms offers a quick and effective method for determining the global optimal point for optimization problems [23]. These algorithms are categorized based on the source of inspiration such as by evolution, by swarms, by physics, by humans, by biology, and by mathematics. Optimizing CNN hyperparameters using metaheuristic-based approaches proves effective; however, there isn't a universally superior solution applicable to all optimization challenges [24,25].

A hybrid metaheuristics-deep learning strategy was presented by the authors of [26] to improve intrusion detection in IoT. Through the integration of powerful metaheuristic algorithms with a group of recurrent neural networks (RNNs), particularly LSTM and GRU models, the system could detect and classify a wide range of threats in IoT. The approach proved to be more accurate and efficient than others through the use of algorithms like fractional derivative mutation and Harris hawk optimization. The main purpose of the study [27] was to enhance the performance of the Gorilla Troops Optimizer (GTO) by employing an algorithm for bird swarms, in order to introduce a new feature selection (FS) strategy. Four IoT-IDS datasets were used to examine the performance of the suggested method against those of other competitive algorithms that are currently in use: NSL-KDD, CICIDS-2017, UNSW-NB15, and BoT-IoT. It was shown by the results of research that the indicated technique thereof preceded over no less than several existing metaheuristic algorithms. The authors of [28], suggested feature selection utilizing the hybrid strategy (DT and LR) in conjunction with GA to categorize the dataset. They used feature extraction to reduce the features that adversely make the system more complex. To optimize the selected ideal characteristics in this study, the efficacy of various metaheuristic algorithms has been compared. The study’s results show that the GWO algorithm was the algorithm with the greatest accuracy attainable, which was 99.44 %. Deep learning was employed by the authors of [29] for building MDLIDS-SSE, a new metaheuristic for intrusion detection in a secured smart environment. This approach involves Z-score normalization for data preprocessing, an arithmetic optimization algorithm-based feature selection, and quantum-controlled particle swarm optimization with a deep wavelet neural network model for intrusion detection and classification. The simulation results show that MDLIDS-SSE performs better than other currently existing intrusion detection methods. Neural Architecture Search (NAS) based on evolutionary computation (EC) which considers the problem of computational complexity and flexibility in design was proposed in [30].

The authors of [31] provide the PSO-based EAEPSO as the effective neural architecture search technique. The network is encoded into the fixed-length latent vectors with the use of an autoencoder. Moreover, the hierarchical fitness evaluation technique is used as a direction to the search by EAEPSO. EAEPSO's competitiveness is demonstrated by experimental findings, which provide low error rates of 2.74 % on CIFAR-10 and 16.17 % on CIFAR-100, respectively, with a significant reduction in computational costs to 2.2 and 4 GPU-days only.

Another study, carried out by the authors of [32], focused on improving CNN performance using the harmony search (HS) method. Additionally, authors in [33] presented models utilizing evolutionary algorithms, demonstrating high efficiency on intricate networks. The authors of [34] proposed the GACNN model, employing a convolution genetic neural network based on random samples, demonstrating improved solutions for uncovering unknown character expressions. Experimental results, conducted on the MNIST dataset, affirm the model's advantages [35], [36], [37]. The authors have determined the advantages of combining CNN with metaheuristics for hyperparameter searching.

CNN hyperparameter optimization is a continuous task. In light of IDS's criticality, significance, and efficacy for IoT networks, this challenge presents an excellent, meaningful, and important research problem.

## Metaheuristic algorithms

Metaheuristic algorithms are used to develop approximate solutions to complex optimization problems. Unlike traditional optimization methods, metaheuristic algorithms do not guarantee finding the global optimum, but they are able to explore large solution spaces efficiently and effectively. They use iterative improvement strategies to search for good solutions.

Some common metaheuristic optimization algorithms include:

### Particle swarm optimization (PSO)

**Fig. 1:**
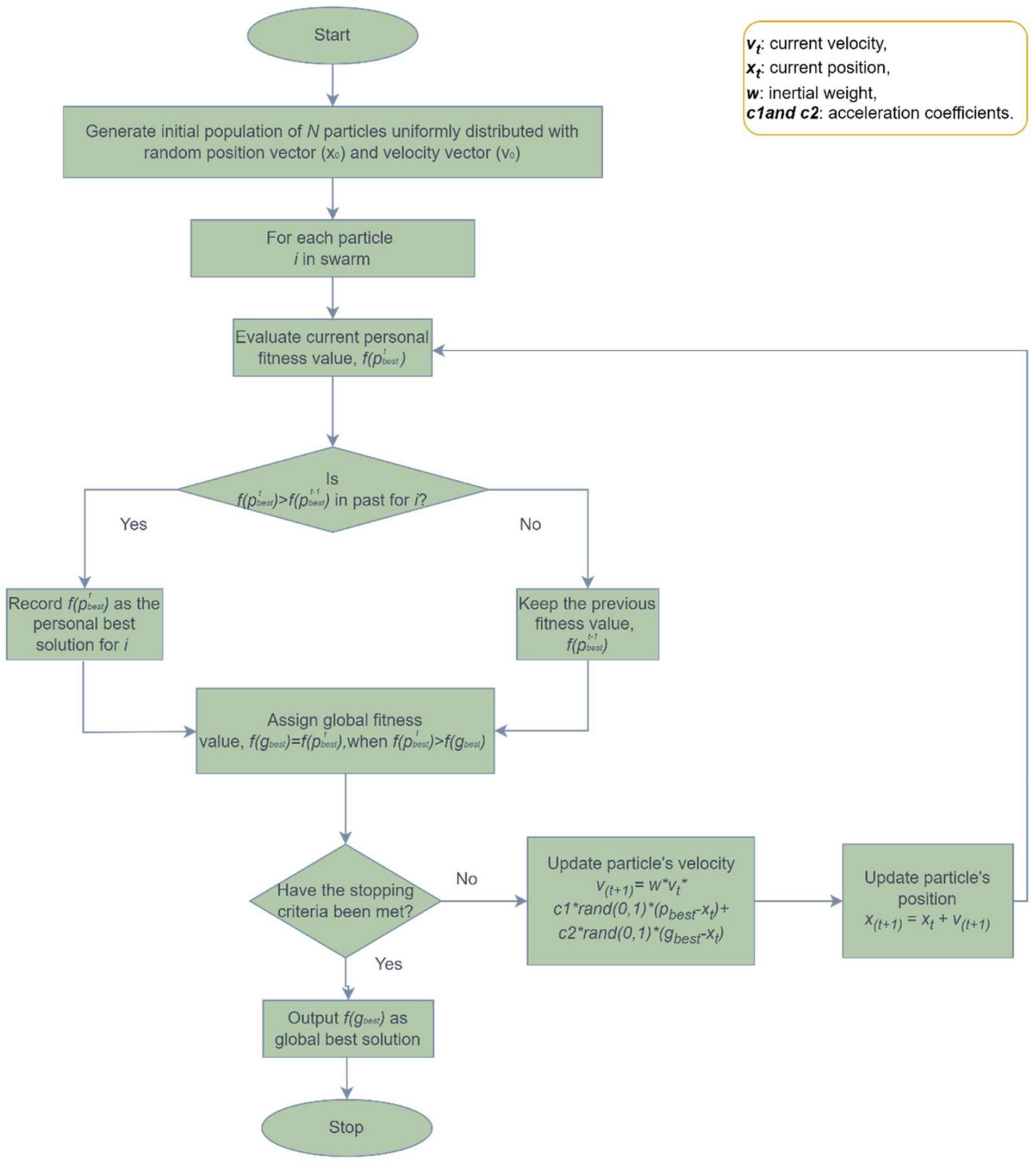
PSO flowchart.

PSO [14], introduced by James Kennedy and Russ Eberhart in 1995, has found applications in diverse search and optimization challenges. This nature-inspired metaheuristic technique mimics the collective behaviour of natural swarms, such as birds, fish, etc. to solve complex problems. In PSO, a swarm comprising of *‘n’* particles interacts and communicates, aiming to discover optimal solutions. Each particle possesses three key vectors: the current position (x_vector), velocity (v_vector), and the self-best solution found so far (p_vector). The swarm explores potential solutions within a defined search area, with particles dynamically representing the range of potential solutions. PSO operates by accelerating particles toward their best-known positions and the overall best position found by any particle, incorporating random weighted accelerations. Throughout iterations, each particle updates its self-best solution (p_best_) and tracks the global best solution (g_best_) within the swarm. By combining individual and collective experiences, particles adjust their positions and velocities, converging toward a promising solution. This iterative process repeats till the global best solution is achieved, effectively addressing the optimization problem. Fig. 1 illustrates the PSO flowchart.

### Genetic algorithm (GA)

**Fig. 2:**
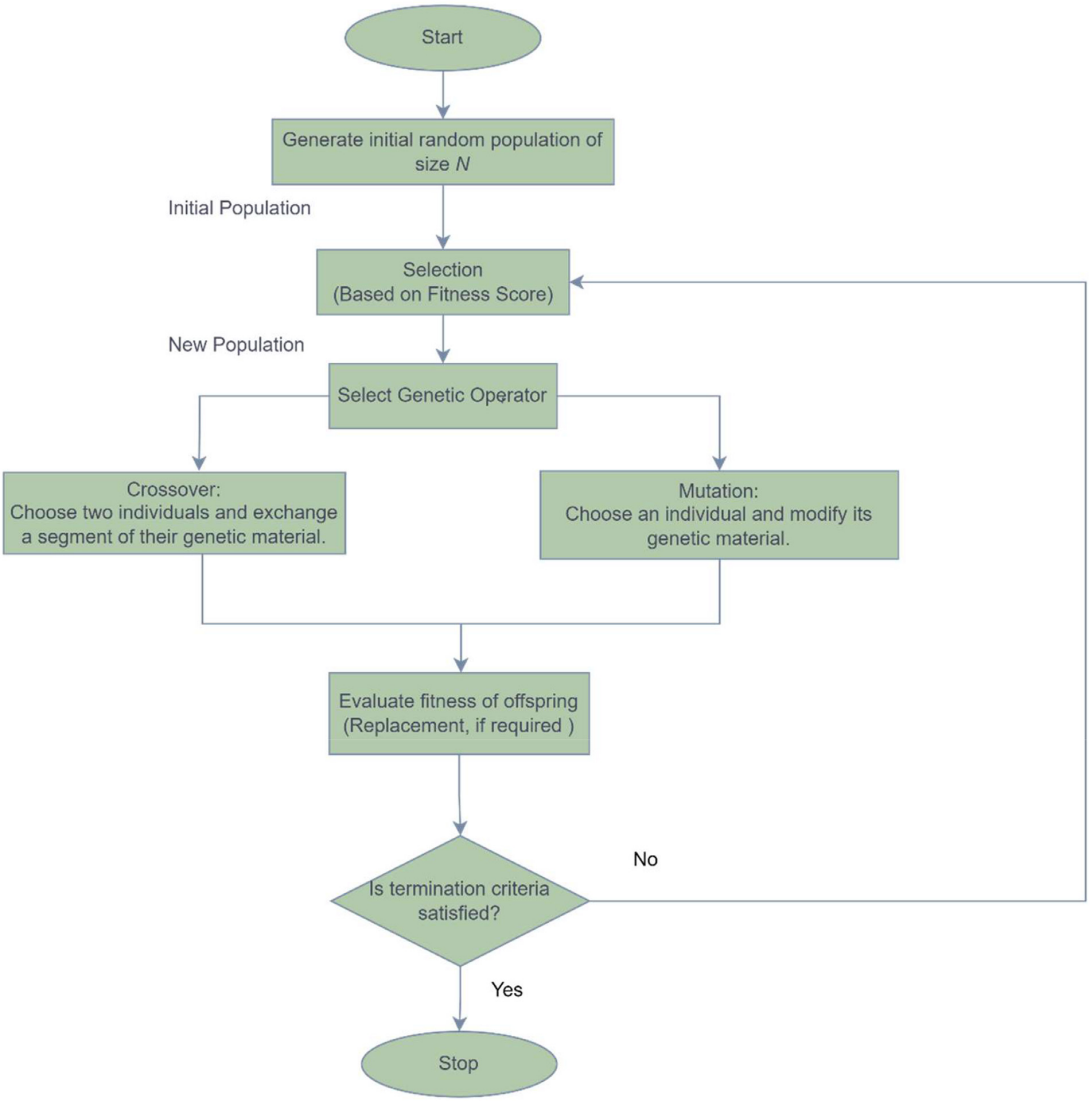
GA flowchart.

GA [15], pioneered by John Holland, is an adaptive heuristic search approach inspired by Charles Darwin's principles of natural selection and genetic mechanisms observed in biological evolution. This algorithm iteratively adjusts a population of individual solutions. In each cycle, individuals with higher fitness scores (problem-specific) from the current population are selected as parents, contributing to the creation of children for the next generation. Across multiple generations, the population progressively refines itself, converging towards an optimal or near-optimal solution. Fig. 2 illustrates the flowchart for the genetic algorithm process.

The genetic algorithm optimization technique typically involves the following steps:

1. Initialization:
  • Establish an initial population of potential solutions (individuals) using chromosomes (such as binary strings or real-valued vectors).
  • Based on how well each individual solves the problem at hand, assign them an initial fitness score.
2. Selection:
  • Apply the objective function to assess the fitness of each member of the population.
  • Choose the individuals who score higher on fitness to serve as parents to reproduce.
  • Rank-based, tournament, and roulette wheel selection are examples of common selection techniques.
3. Crossover:
  • Crossover is the process of exchanging genetic material between specific pairings of parents to produce offspring.
  • Common strategies for performing crossover include one-point, two-point, and uniform crossover.
4. Mutation:
  • Mutation introduces random changes in the genetic material of certain children to ensure genetic diversity.
  • Mutation might help avoid an early convergence to a suboptimal solution by introducing diversity into the population.
5. Evaluation:
  • Determine the offspring's fitness and consider replacing the previous population with a new one that includes parents, crossover, and mutation offspring.
  • Depending on the replacement technique, either all of the population will be replaced or just a section will be substituted.
6. Termination:
  • Verify whether a requirement for termination is satisfied, such as completing a predetermined computation time limit, reaching a maximum number of generations, or achieving a satisfying result.
  • The algorithm halts if the termination requirement is satisfied; if not, it repeats the selection phase and the evolution process goes on.

### Grey wolf optimization (GWO)

**Fig. 3:**
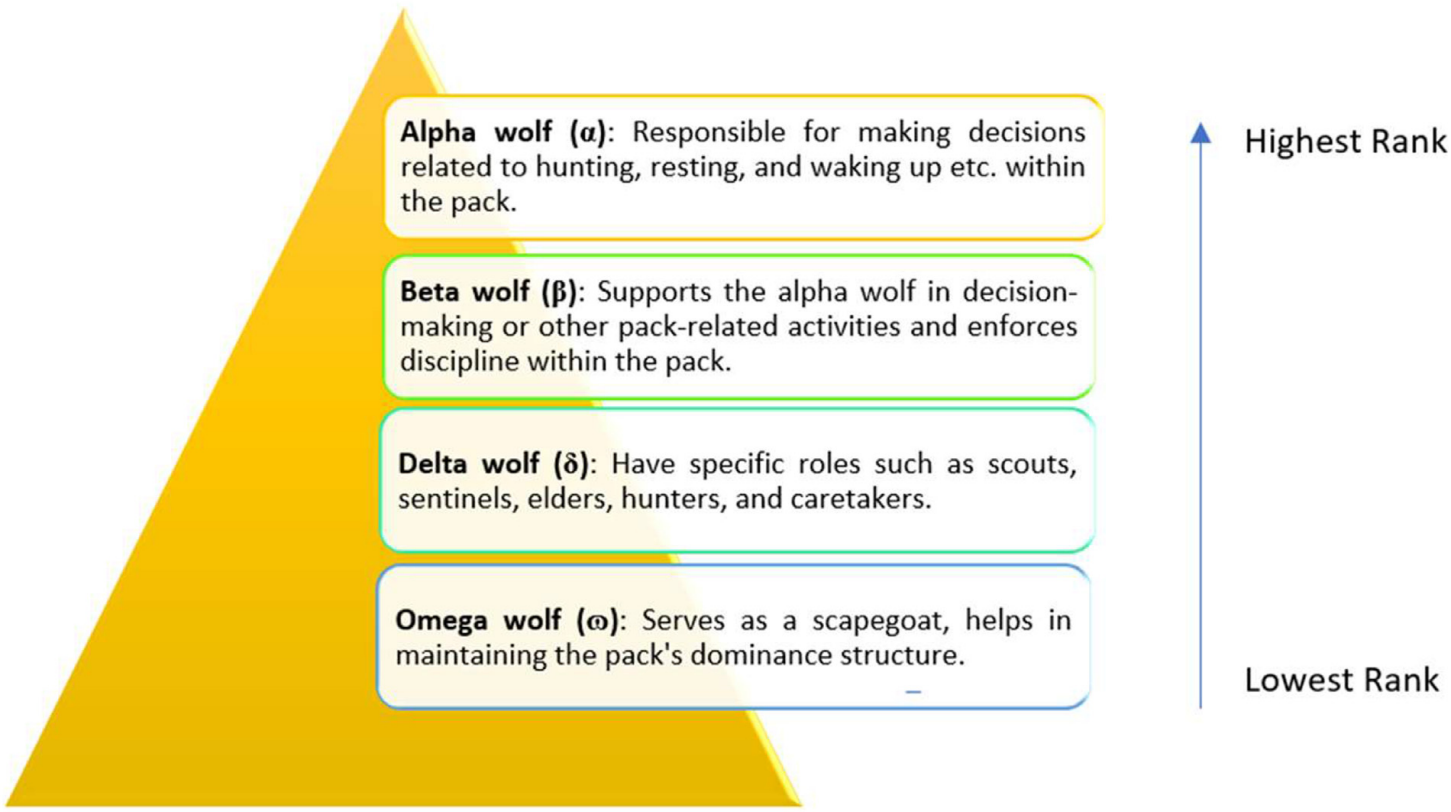
Hierarchy level of grey wolves in the pack.

In 2014, Mirjaliali Mohammad and Lewis introduced the metaheuristic approach known as GWO [16]. It is influenced by the social structure and methods of hunting of grey wolves found in the wild. In their natural habitat, grey wolves tend to form packs consisting of 5–12 members, showcasing a well-defined social hierarchy, as illustrated in Fig. 3.

Each wolf in GWO represents a potential solution and occupies a distinct place in the solution space. The optimization process is driven by the fittest solution, designated as alpha (α), along with the second and third-best solutions identified as beta (β) and delta (δ), respectively. The remaining candidate solutions are denoted as omega (ɷ). The algorithm simulates the cooperative hunting style of wolves. Grey wolves use an encirclement technique around their prey during hunting. This encircling behaviour is mathematically expressed through specific equations:

(1) $$\vec{D}=|\vec{C}\;.\left({\vec{X}}_{p}\left(t\right)-\;\vec{X}\left(t\right)\right|$$

(2) $$\vec{X}\left(t+1\right)=\;{\vec{X}}_{p}\left(t\right)\;-\vec{A}.\vec{D}$$

Where, *‘t’*: current iteration,

$\vec{A}=\;2\vec{a}.\;{\vec{r}}_{1}-\;\vec{a}\;and\;\vec{C}=2.{\vec{r}}_{2}$: coefficient vectors,

${\vec{X}}_{p}\left(t\right)$: position vector (prey),

${\vec{X}}_{g}\left(t\right)$: position vector (grey wolf), and

${\vec{r}}_{1},\;{\vec{r}}_{2}:$ random vectors within the range [0,1].

Over the course of the iterations, the components of $\vec{a}\;$progressively drop from 2 to 0.

The provided equations serve the purpose of updating a grey wolf's position randomly in the space surrounding the prey.

**Fig. 4:**
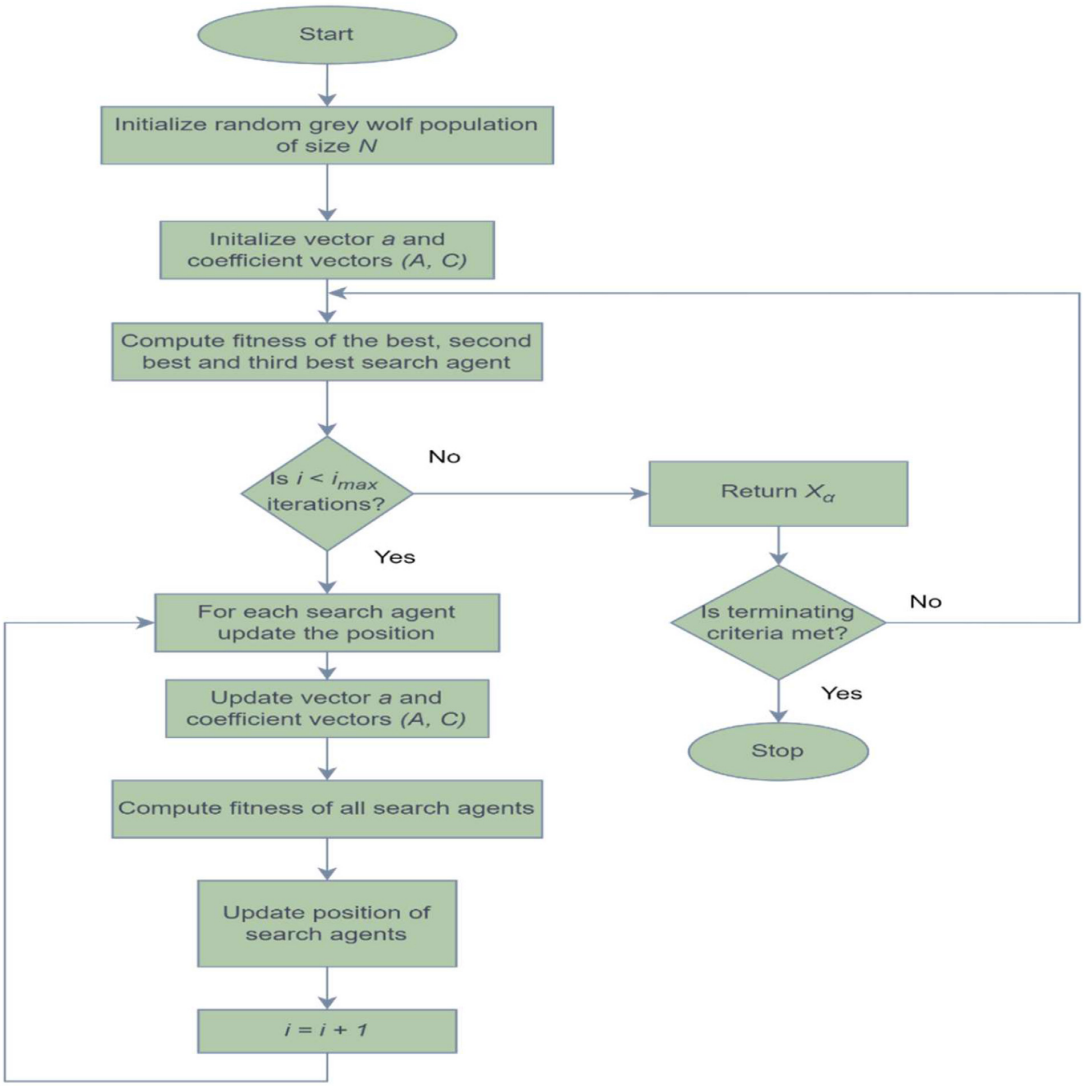
GWO flowchart.

As the head of the pack, the alpha plays a vital role in directing the hunting process. The alpha, beta, and delta wolves are supposed to have an extensive understanding of the possible prey's location. As a result, the top three best options found so far are conserved. The positions of the other search agents are then modified in accordance with the known positions of these high-achieving search agents. To put it simply, other wolves update their positions randomly around this projected prey location while alpha, beta, and delta estimate the prey's position. The hunting process concludes with the wolves initiating an attack on the prey when it remains stationary. The wolves disperse to explore various areas (exploration) for prey and converge when potential solutions are identified (exploitation). By mimicking these behaviours, the algorithm gradually converges towards the best possible solution for the given problem. Fig. 4 illustrates the GWO flowchart, depicting these steps in the optimization process.

### Ant lion optimization (ALO)

An ant lion's hunting strategy is modelled by ant lion optimization (ALO) [17], a nature-inspired metaheuristic optimization method. It was created by Seyedali Mirjalili in 2015, and a variety of optimization issues have been effectively solved with it.

The optimization process of ALO involves the following steps:

• Ant Movement

This phase initiates the optimization process where individual search agents (analogous to ants) are assigned a random position within the search space. These agents then explore their surroundings by moving in random directions for a random distance. This exploration continues till the agent reaches the edge of the search space or encounters a trap (ant lion trap), all the while attempting to discover promising solutions to the problem.

• Trap Construction

The traps are constructed by the ant lions based on their fitness levels. Agents with higher fitness construct more intricate "traps" in their respective locations, indicating areas of the search space with potentially optimal solutions.

• Selection using Roulette Wheel Operator

A selection process based on the "roulette wheel" prioritizes agents with higher fitness for reproduction. This process ensures that desirable features, represented by higher fitness values, are passed on to the next generation. By favouring fitter individuals, the algorithm converges towards regions with more promising solutions.

• Trap Activation and Capturing Prey

When an ant enters a trap, ant lions react by shooting sands outward from the trap’s center thereby making it difficult for the trapped ant to escape. This method is similar to how actual ant lions catch prey in their sandpits. The successful capture of an ant indicates the discovery of a potential optimal solution.

• Consumption and Trap Rebuilding

After capturing an ant, the ant lion consumes it for energy. This energy is used to reinforce its trap-building behaviour and continue searching for improved solutions. Ant lions update their positions based on the hunted ants, enhancing their strategies to catch new prey, leading to further optimization.

• Population Evolution

Some ant lions are replaced with captured prey, allowing the population to evolve. Fitter ant lions, successful in capturing prey, contribute to the next generation. This step ensures that the population contains individuals with advantageous traits, promoting continuous optimization over iterations.

• Extraction of the Best Solution

After the optimization process, the best solution found by the ant lions is extracted. This solution represents the optimal or near-optimal solution to the problem. This step is essential as it identifies the output of the optimization process, providing the user with the best solution discovered by the algorithm.

### Aquila optimization (AO)

AO [18,38], is a population-based metaheuristic algorithm inspired by the hunting behaviours of the Aquila bird in nature. Renowned for its remarkable visual acuity and hunting prowess, the Aquila bird efficiently captures prey.

The AO algorithm starts by initializing a population of possible solutions, which are represented as individuals within the population. In each iteration of the algorithm, every individual in the population evaluates its fitness based on a problem-specific fitness function. This function quantifies how well the solution satisfies the optimization criteria. The individuals reposition themselves depending on both their previous positions in the population and the positions of other individuals. Eventually, the AO will stop when a given stopping criterion is met. At that moment, its iterative searching was done. Diversification (exploration) and intensification (exploitation) are the two phases on which the algorithm operates. The diversification phase involves incorporation of random operators to explore various parts of the search space. The algorithm's intensification phase's objective is to search for the best possible solution within the search space by advancing its search strategy. The algorithm seeks to find the best individual(s) in the population and then provides the ideal solution to the optimization problem.

## Materials and methods

### Material

For experimentation purpose, we employed a computing infrastructure having a Windows 10 Operating System with an AMD Ryzen 5 (4500U) processor, AMD Radeon graphics, 2.38 GHz clock speed, 256 GB SSD, and 8 GB RAM. To generate an IoT-specific dataset, we utilized the Contiki-OS Cooja Simulator Furthermore, we utilized Jupyter Notebook to execute our deep learning models, leveraging its capabilities for seamless experimentation and analysis.

### Method

**Fig. 5:**
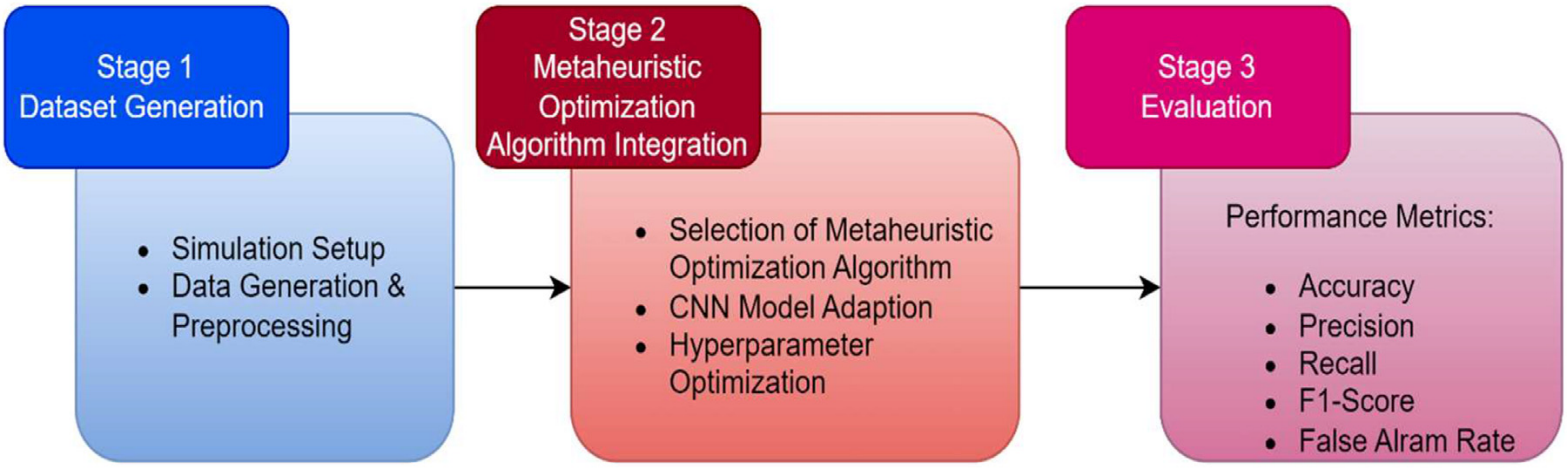
Proposed methodology.

In the subsequent section, we present a methodology for creating a malicious dataset using the Cooja Simulator, integrating various metaheuristic algorithms into a dedicated CNN model, and evaluating the performance of these models. The dataset, generated in-house, will serve as the basis for training and testing the different models. Fig. 5 illustrates the methodology employed for this study.

Below is a comprehensive breakdown of each stage:

#### Stage 1: Dataset generation

During the "Dataset Generation" stage, we perform the following steps:

• Simulation Setup:

The simulation setup step involves configuring the Contiki Cooja Simulator to replicate a real-world IoT scenario affected by blackhole, sinkhole, flooding, and version number attacks. To introduce variability, the positioning of the nodes in the network was randomized, ensuring accurate replication of a real-world IoT scenario. Specific simulation parameters were meticulously defined and set to execute attacks in an IoT environment as shown in Table 1.

**Table 1:** Simulation parameters.

Simulator Parameter	Value
Root node	1
Sender nodes (Normal)	2, 3, 5, 6, 7, 9, 10, 11, 13, 14
Sender nodes (Malicious)	4, 8, 12, 15
Radio Medium	UDGM: Distance Loss
Transmission Range	100 m
Interface Range	50 m
Mote type	Sky Mote: 1, 2, 3, 4, 5, 6, 7, 8, 9, 10, 11, 13, 14, 15. Z1 Mote: 12
Mote startup delay(ms)	1000 ns
Random Seed	123,456

**Fig. 6:**
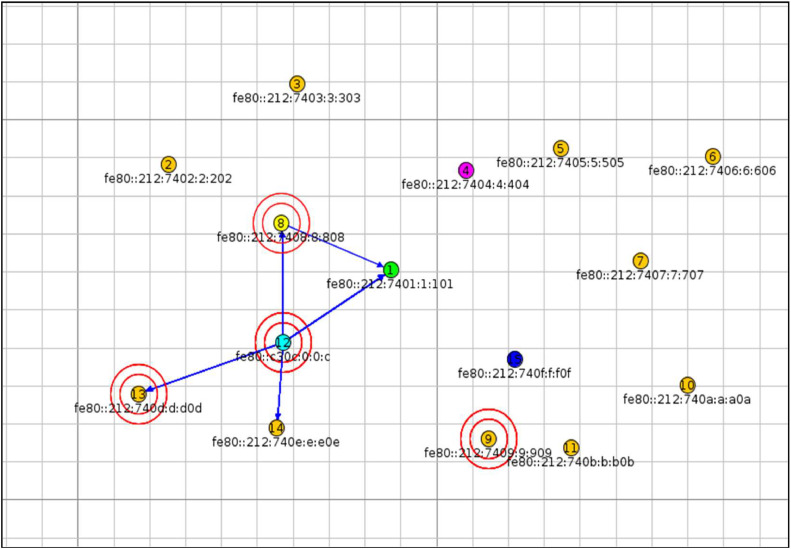
Network topology considered for launching attacks in Cooja Simulator.

The network topology employed for executing the attacks, including blackhole, sinkhole, flooding, and version number attacks, is depicted in Fig. 6. Specifically, nodes 4, 8, 12, and 15 are allocated for carrying out the blackhole, sinkhole, flooding, and version number attacks, respectively.

In the context of IoT, a blackhole attack occurs when a malicious node falsely advertises itself as having the shortest or best path to a destination node. When data packets are sent to the malicious node, it drops or consumes the packets without forwarding them to the intended destination. This disrupts communication between legitimate nodes, leading to network performance degradation or even complete network failure. Whereas, in sinkhole attack, an attacker manipulates routing information, diverting data packets toward the sinkhole. This malicious node can then eavesdrop on sensitive information or launch further attacks, compromising the confidentiality and integrity of IoT communications. In a flooding attack, the network is disturbed by a malicious node that sends in an unreasonably large number of packets that use up network resources and bandwidth. In other words, this attack impedes normal communication, thus there is a denial of service for legitimate nodes. Moreover, a version number attack leverages vulnerabilities in software versions that have not yet been patched or are old for the purpose of targeting IoT devices. Such attacks can result in data breaches, unauthorized access, and device manipulation.

• Data Generation and Data Preprocessing

The data generation process commences after the simulation setup step. Here, using the Radio Message Tool, data is captured in a .pcap file labeled as radiolog-1699205267986.pcap in our case.

The Wireshark tool is then used to help with the analysis and extraction of the data from the .pcap file. The extracted data is stored in a .csv file. Following that, the collected data undergoes data preprocessing, a crucial step intended for cleaning and standardizing the dataset to ensure consistency and compatibility. This step entails removing noise and irrelevant information from the data. Data preprocessing prepares the data for further analysis and model training. The generated dataset comprises of features, including packet number, timestamp, source and destination IPv6 addresses, communication protocol, packet size, ICMPv6-related information, as well as corresponding details of the simulated network.

#### Stage 2: Metaheuristic algorithm integration

In this stage, the metaheuristic algorithms are systematically integrated with CNN by choosing an appropriate algorithm, followed by modifying the CNN model, and tuning hyperparameters. The process of integration is aimed at increasing the efficacy of the IDS, thus ensuring a well-functioning security system for the complex and constantly evolving IoT networks.

• Algorithm Selection: The primary step involves selecting the most appropriate metaheuristic algorithm for IoT intrusion detection tasks. Specifically, here, PSO, GA, GWO, ALO, and AO are selected because of their suitability and effectiveness in other studies in handling the complexities of IoT environments. Besides their capability to work together with CNNs, their ability to optimize hyperparameters for machine and deep learning models and the potential to improve the effectiveness of intrusion detection systems in IoT environments were also crucial factors for consideration.
• CNN Model Adaptation: At this step, CNN's architecture is adapted to better suit the characteristics of IoT networks. The given modification guarantees that the suggested CNN model can process IoT-specific data efficiently, allowing it to work with the chosen metaheuristic algorithm.
• Hyperparameter Optimization: At this step, the most suitable metaheuristic algorithm is combined with the modified CNN model. The number of units, kernel size, pool size, and strides—a step size are the hyperparameters to be tuned. Optimization is crucial since it modifies the CNN model, enhancing its efficacy in IoT network intrusion detection tasks. The combination of metaheuristic algorithms with CNN facilitates a methodical examination of the hyperparameter space to determine the ideal settings that enhance the overall efficiency and accuracy of the CNN model in intrusion detection tasks.

**Fig. 7:**
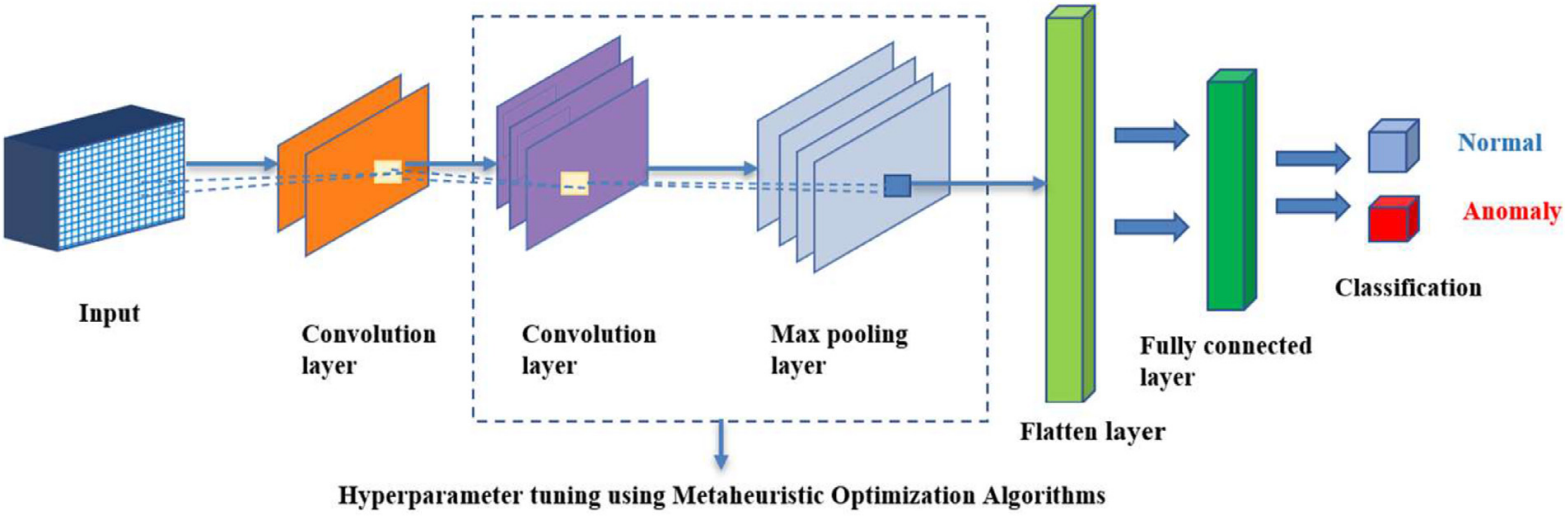
A general metaheuristic optimizer-CNN combination architecture.

In this study, we employed a particular architectural design that combines metaheuristic algorithms with CNN, as shown in Fig. 7. This architecture utilized a sequential model comprising two convolution layers, a max pooling layer, and a flatten layer, followed by an output layer. A fitness function was employed, to compute values for parameters such as the number of units, kernel size, pool size, and strides through metaheuristic algorithms, ensuring the discovery of the most optimal system configurations. The optimization process involved refining these parameters to obtain the best fitness values. Subsequently, based on the returned optimal fitness values and corresponding parameter solutions, unique metaheuristic-CNN combination models were constructed. These models were meticulously trained leading to the development of highly effective metaheuristic-CNN hybrid models tailored for IoT intrusion detection tasks.

#### Stage 3: Evaluation

After the models undergo training, an assessment of their performance is conducted using the testing dataset for each attack scenario. The efficacy of different models, namely CNN-PSO, CNN-GA, CNN-GWO, CNN-ALO, and CNN-AO is measured using a variety of metrics to determine their ability to perform well on new, unseen data and generalize effectively. The following metrics have been used to evaluate the performance of these models:

➢ Accuracy: It represents the proportion of correctly predicted instances to the total number of instances in the given dataset.
➢ Precision: It tells us what percentage of the positive predictions the model has predicted correctly by dividing these by all the total predicted positives.
➢ Recall or Sensitivity: It is obtained by dividing correctly predicted positives by all the actual positives in the given dataset.
➢ F1-score: It offers an appropriate trade-off between precision and recall. It represents harmonic mean of precision and recall, thus, offering an overall assessment of the model's competence.
➢ False Alarm Rate: This is the proportion of false positive results to actual negatives among all predictions. A lower false alarm rate indicates the model's competence in avoiding a misclassification of negative instances as positive, thus, validating the model's reliability in detecting true negatives.

## Method validation

This study employed a self-generated malicious dataset specifically designed to imitate intrusion scenarios within IoT network. This dataset formed the basis for further analysis of different metaheuristic algorithms integrated with CNN, revealing which of the combinations is more appropriate for the identification of significant vulnerabilities in IoT networks. An evaluation of the performance of CNN-PSO, CNN-GA, CNN-GWO, CNN-ALO, and CNN-AO models for the self-generated malicious dataset as well as other models is given in Table 2.

**Table 2:** Evaluation results of CNN-PSO, CNN-GA, CNN-GWO, CNN-ALO, and CNN-AO models and others.

Dataset	Model	Population Size	Accuracy (%)	Precision (%)	Recall (%)	F1-score (%)	FAR (%)
**Self-Generated Malicious Dataset**	Naïve Bayes	–	80.77	74.32	88.20	80.67	25.41
	Random Forest	–	88.40	97.74	76.26	85.67	1.47
	CNN-PSO	10	88.41	97.77	76.26	85.69	1.44
	CNN-GA	50	77.79	70.11	89.16	78.50	31.68
	CNN-GWO	10	77.64	70.04	88.83	78.32	31.68
	CNN-ALO	10	94.86	99.18	89.44	94.05	0.61
	**CNN-AO**[38]	**10**	**95.36**	**99.30**	**90.45**	**94.66**	**0.53**

As shown in Table 2, this study has offered insights into the effectiveness of the combination of metaheuristic and CNN techniques in intrusion detection in IoT networks.

Evidently, the CNN-AO model has been proven the most successful IDS and has demonstrated remarkable result with an accuracy of 95.36 %, precision of 99.30 %, recall of 90.45 %, F1-score of 94.66 %, and a notably low False Alarm Rate (FAR) of 0.53 %. This implies the CNN-AO has acceptable stability concerning the detection of attacks in IoT networks.

Unlike other metaheuristic-CNN combinations, i.e., CNN-ALO and CNN-PSO, too have demonstrated the desired results, inferring their relevance in intrusion detection tasks. On the contrary, the CNN-GWO and the CNN-GA models offer lower accuracy and precision results, which in turn indicates the significance of selecting the most suitable metaheuristic algorithms for effective integration into CNNs.

## Conclusion

In conclusion, this research highlights the importance of cybersecurity especially given the growing challenges within the IoT landscape. The present work entails important findings into the field of IoT security, with rigorous design of the dataset used. This custom dataset, which is built using Contiki Cooja Simulator, aims to mimic real-world IoT scenario impacted by blackhole, sinkhole, flooding and version number attacks. The self-generated dataset for this research acts as a foundation, which helps to examine a variety of frameworks. Apart from this, the integration of several metaheuristic algorithms such as PSO, GA, GWO, ALO, and AO with CNNs was also one of the main considerations during this research. Table 2 sets out the results, which demonstrate the efficiency of these combinations in addressing most attacks on IoT systems. On the other hand, the CNN-AO model showed excellent performance in terms of very high accuracy, precision, recall and F1-score and a low false alarm rate.

This study leads to diverse avenues for future research. Exploring novel machine learning techniques and advanced deep learning architectures could further enhance the accuracy and efficiency of intrusion detection systems in IoT environments. Additionally, real-time intrusion detection mechanisms and tailored anomaly detection algorithms represent promising areas for promptly identifying emerging threats. Moreover, extending our research to incorporate edge computing solutions and federated learning techniques could enhance the scalability and privacy aspects of IoT security. Collaborative efforts between researchers and industry experts are crucial for adapting security strategies continually, ensuring a robust defense against the ever-evolving cyber threats within the dynamic IoT landscape.

## Funding information

We thank for receiving the funding for this from RSF (Research Support Funding) from Symbiosis Institute of Technology, Symbiosis International (Deemed University) (SIU), Lavale Campus, Pune, Maharashtra, 412115, India.

## Ethics statement

The paper reflects the authors' own research and analysis in a truthful and complete manner.

## CRediT authorship contribution statement

**Vandana Choudhary:** Conceptualization, Investigation, Writing – original draft. **Sarvesh Tanwar:** Supervision, Writing – review & editing. **Tanupriya Choudhury:** Supervision, Writing – review & editing. **Ketan Kotecha:** Supervision, Writing – review & editing.

## Declaration of Competing Interest

The authors declare that they have no known competing financial interests or personal relationships that could have appeared to influence the work reported in this paper.

## Data Availability

Data will be made available on request.
